# Through history to growth dynamics: deciphering the evolution of spatial networks

**DOI:** 10.1038/s41598-022-24656-x

**Published:** 2022-11-27

**Authors:** Stanisław Żukowski, Piotr Morawiecki, Hansjörg Seybold, Piotr Szymczak

**Affiliations:** 1 Institute of Theoretical Physics, Faculty of Physics, University of Warsaw, Warsaw, Poland; 2 Laboratoire Matière et Systèmes Complexes (MSC), UMR 7057, CNRS & Université Paris Cité, Paris, France; 3 Department of Mathematical Sciences, University of Bath, Bath, UK; 4 Department of Environmental System Science, ETH Zürich, Zürich, Switzerland

**Keywords:** Physics, Fluid dynamics, Statistical physics, thermodynamics and nonlinear dynamics

## Abstract

Many ramified, network-like patterns in nature, such as river networks or blood vessels, form as a result of unstable growth of moving boundaries in an external diffusive field. Here, we pose the inverse problem for the network growth—can the growth dynamics be inferred from the analysis of the final pattern? We show that by evolving the network backward in time one can not only reconstruct the growth rules but also get an insight into the conditions under which branch splitting occurs. Determining the growth rules from a single snapshot in time is particularly important for growth processes so slow that they cannot be directly observed, such as growth of river networks and deltas or cave passages. We apply this approach to analyze the growth of a real river network in Vermont, USA. We determine its growth rule and argue that branch splitting events are triggered by an increase in the tip growth velocity.

## Introduction

**Figure 1:**
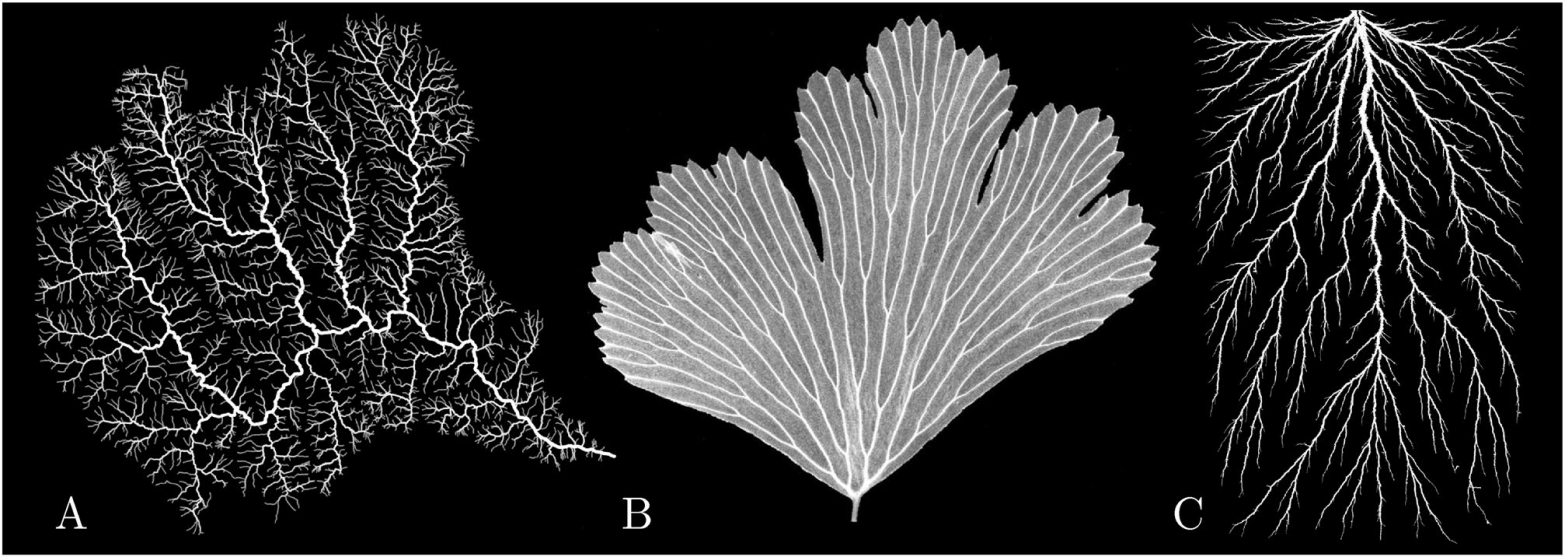
Examples of branched spatial networks in nature: (**A**) White River basin in Vermont, USA^7^ (**B**) Adiantum leaf (photo: Jim Mattsson, Simon Fraser University, by permission) (**C**) Lichtenberg figure^8^.

Many of the natural patterns are in the form of branched networks: from river networks or cave conduits, mineral dendrites, and viscous fingering patterns to biological systems such as blood vessels or leaf venation^1,2^ (Fig. 1). The physical forces driving their growth vary from system to system: erosion, diffusion, and thermal conduction, to name just a few^3^. Despite these differences, there are many features which these networks share, which suggests a common underlying growth mechanism^4–6^.

A prominent feature of network growth is the close coupling between geometry and dynamics. The field driving the growth and the network co-evolve in time, as the evolving network changes the boundary conditions for the field. Evolving the network in response to the surrounding field involves two major processes: (i) extension of the branches and (ii) bifurcation of a tip into two (or more) branches. To understand the co-evolution of the network and the field, it is important to understand how the extension and bifurcation processes are linked to the characteristics of the driving fields such as the gradient of the field in the vicinity of the tip. Once the *growth rules* are known, we can predict the evolution of the network geometry based on its configuration at an earlier time.

Such procedure has been applied to river networks^9–13^, river deltas^14^, viscous fingers^15^, discharge trees^16^ or coral growth^17^, leading to network geometries qualitatively and quantitatively similar to those observed in nature. However, in many practical cases, the details of the growth rules are unknown. If, in addition, the temporal evolution of the pattern is exceedingly slow and can only be observed at a single instance of time, determining growth rules from a single snapshot in time becomes a necessity. This gives rise to the following question: can we deduce the growth rules from an instantaneous snapshot of the network geometry? In fact, even a single snapshot of a network configuration contains information about its growth history, which is inherently linked to growth dynamics.

For example, streets in cities are historically built in succession—the oldest being the longest and going through the whole city, and the younger extending from the first ones. To extract historical information from city maps, Lagesse et al.^18^ used a multiscale approach exploiting local geometry and alignment at road crossings.

Geological systems, such as river networks, evolve over even longer time scales. They extend through erosion at the channel heads at a rate of less than a few millimeters per year^19^. One idea for deciphering the growth law in these systems is through a backward-forward approach. First, the network is grown backward in time using a parameterization of the growth rule with an initial set of parameters, and then it is grown forward in time again. Analyzing the correlation of the flux coming to the tips and the orientation of the branches after the backward-forward step, one can determine the optimal set of parameters that best replicates the initial network geometry^11^.

**Figure 2:**
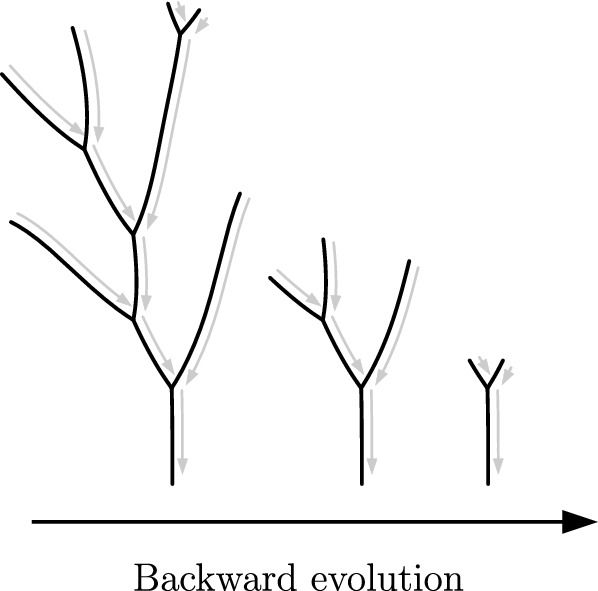
The backward evolution of a network. Consider a network of unknown growth dynamics. We postulate a certain growth rule and use it to evolve the network backward in time. During backtracking, we collect metrics to estimate the fitness of different growth rules and select the best.

In this paper, we present a comprehensive method to extract growth rules from a single snapshot of the network geometry—the Backward Evolution Algorithm (BEA) (Fig. 2). Within the BEA approach, we probe the space of possible growth rules linking the extension of the network with the gradient of the driving field. We then apply these growth rules backward in time, backtracking the evolution of the network completely down to its seeds. While backtracking, we collect multiple metrics to quantitatively estimate the fitness of a given growth rule and select the best. We validate this approach on synthetic data for networks grown in a diffusive field, demonstrating that we can not only successfully determine the growth rules of the branches but also assess the conditions under which branch splitting occurs. The method thus allows for a thorough analysis of the patterns and provides a glimpse into a previously inaccessible growth history.

## The model

We begin by introducing a specific family of growth processes, namely, growth driven by a diffusive field. Here, the field is coupled to the network through the boundary conditions, as the branches absorb the field fluxes. Many natural growth processes can be described by such a system, for example, the formation of blood vasculature^20^, dissolution patterns in porous media^21^, river networks^10,22^ or electric breakdowns^23^.

If the internal dynamics of the field is fast compared to the evolution of the network, the field can be assumed to be quasi-static. This considerably simplifies modeling of the growth process as the diffusion equation reduces to the Poisson or Laplace equation for the driving field. Essentially, the equation governing the field can be derived from Fick’s law, linking the flux ($\vec {J}$) to the gradient of the field ($\nabla \phi$):

1 $$\begin{aligned} \vec {J}=-\kappa \nabla \phi \end{aligned}$$

with a respective transport coefficient $\kappa$. Combining (1) with the conservation of flux ($\nabla \cdot \vec {J} = P$) results in the Poisson equation:

2 $$\begin{aligned} \Delta \phi = -\frac{P}{\kappa } \,, \end{aligned}$$

where *P* is a source term. For the special case $P=0$ we recover the celebrated problem of Laplacian growth^3,24^, in which an interface between two immiscible phases moves with a velocity proportional to the gradient of the harmonic field:

3 $$\begin{aligned} \vec {v} \propto \nabla \phi \,. \end{aligned}$$

If the interface is an isoline of the field and the growth rate is proportional to the field gradient, small perturbations of the interface have a tendency to grow. At short wavelengths, the growth of the interface is stabilized by regularization mechanisms such as surface tension or kinetic undercooling^25^. Otherwise, the flux of the field concentrating on the tips favors very thin fingers and leads to their infinitely fast growth—the so-called ultraviolet catastrophe^26^.

The longer wavelengths are generally unstable and have a tendency to grow and eventually transform into fingers^27–31^. There are two main processes responsible for the pattern formation in these systems: (i) screening between the nearby branches mediated by the harmonic field, which results in an increased growth rate of the longer branches and suppression of growth of the shorter ones, and (ii) tip splitting, when the branch bifurcates, giving rise to a pair of daughter branches. The interplay of these two processes leads to the appearance of a highly ramified hierarchical network-like structure.

Growth dynamics of a similar type underlies a wide range of different processes, including formation of the lungs^32–34^, bacterial colony growth^35^, cave formation^21,36^, metallic dendrite formation in electrochemical deposition^37,38^, dielectric breakdown^23^, discharge trees^16^, combustion fingers^39^, tributary and distributary channel formation in river networks^9,14^, dendritic patterns in superconducting films^40^, leaf venation^41^, or blood vasculature formation^20,42,43^.

### The thin finger model

Not every moving boundary problem leads to the emergence of a spatial network. Spatial transport networks have distinct branches, the widths of which are much smaller than the distances between them. They are also characterized by well-defined bifurcation points in which one branch splits into two. It is thus natural to describe the growth of the network in frames of the thin finger model (TFM), which approximates the growing fingers as lines that extend only in length^44–46^. As noted in Ref^44^, such an approach provides also another method of regularizing the Laplacian growth at short wavelengths, without the need of introducing the surface tension. This model has been used for the analysis of fingered growth in both the Laplacian^45–48^ and Poissonian fields^49,50^.

Removing the ultraviolet catastrophe through the thin finger approximation comes at a cost. First, the field gradient at the tip becomes singular, diverging like $r^{-1/2}$ in the vicinity of the tip. In polar coordinates (where the direction of the tangent to the finger at the tip sets $\theta = 0$—see *Supplemental Information*, Fig. S1A) the field near the tip can be expanded^51,52^:

4 $$\begin{aligned} \phi (r,\theta )=a_1r^{1/2}\cos \frac{\theta }{2}+a_2r \sin \theta +a_3 r^{3/2} \cos \frac{3\theta }{2}+\mathscr {O}\left( r^2\right) \,, \end{aligned}$$

where the coefficients $a_i$ depend on the boundary conditions far from the finger tip. Equation (4) holds also for the case of Poisson fields, as in a small area around the tip the flux contribution from the source term is negligible compared to the flux from the regions away from the tip. Each of the leading coefficients $a_i$ in Eq. (4) has a clear physical interpretation^10^ (*Supplemental Information*, Fig. S1).

First, the coefficient $a_1$ is linked to the total flux over a small circle of radius $r_0$ around the tip (*Supplemental Information*, Fig. S1E–F):

5 $$\begin{aligned} J|_{r_0} = \oint _{r_0} (-\kappa \nabla \phi ) \cdot \hat{n} \ \text {d}l = 2 \kappa a_1 r_0^{1/2} + \mathscr {O}\left( r_0^{3/2}\right) \,, \end{aligned}$$

where $r_0$ is the typical width of the finger.

The coefficient $a_2$ is related to the field asymmetry with respect to the finger growth direction (*Supplemental Information*, Fig. S1G). With the positive $a_2$ flux of the field is larger on the right side of the tip and with the negative $a_2$ on the left. However, the finger grows in the direction of the largest flux and, as a result, it turns in such a way that $a_2$ always vanishes (principle of local symmetry^11^).

Finally, $a_3$ is related to the bimodality of the driving field in the neighborhood of the tip. If we consider a circle of radius $r_\text {B}$ around the tip and study the field as a function of the angle $\theta$, we notice that with a fixed $a_1>0$ and $a_2=a_3=0$ the field has a single maximum at $\theta = 0$ (*Supplemental Information*, Fig. S1E–F). Now, if we take $a_3<0$, then the smaller it is, the flatter the maximum becomes. Eventually, when the second derivative of $\phi$ becomes negative, which corresponds to the negative value of $a_3/a_1$:

6 $$\begin{aligned} \frac{\partial ^2 \phi }{\partial \theta ^2}\big |_{\theta =0, r= r_\text {B} }< 0 \ \ \implies \ \ a_3/a_1 < -\frac{1}{9 r_\text {B}} \,, \end{aligned}$$

the field becomes bimodal and there appear two maxima of the flux at $\pm \theta _0$ (*Supplemental Information*, Fig. S1H). Note that the threshold value of $a_3/a_1$ depends on the distance $r_\text {B}$ from the tip at which the field is analyzed. For sufficiently small $r_\text {B}$ the field profile is always symmetric with one maximum at $\theta =0$^13^.

### Growth rules

The growth velocity in classical Laplacian growth is proportional to the field gradient. A widely used extension of this rule assumes a power-law relation between the flux into the tip and the growth velocity^23,53^:

7 $$\begin{aligned} v \propto J^\eta \propto |\nabla \phi |^\eta \,, \end{aligned}$$

where $\eta$ is a specific growth exponent. Recalling Eq. (5), we have:

8 $$\begin{aligned} v = \sigma a_1^\eta \,. \end{aligned}$$

where $\sigma$ is the proportionality constant linking flux with the growth rate of the tip. The value of the growth exponent, $\eta$, strongly affects the competition between the fingers^45,46,54^ and hence, as shown later, results in qualitatively different network geometries (Fig. 4).

While the moving boundary problem in its continuous version has an inherent instability, which splits one finger into two daughter branches depending on the finger width and speed^55^, in the TFM tip splitting is not an intrinsic part of the dynamics^56^ and needs to be introduced by hand, based on certain criteria. Two different splitting criteria can be found in the literature—the velocity criterion^15,28^ and the bimodality criterion^10,17^.

The first is based on the observation that the instability wavelength ($\lambda$) decreases with increasing front propagation velocity^25,28^. As the finger accelerates at some point $\lambda$ becomes smaller than its width, and the finger becomes destabilized. Such a criterion can be straightforwardly implemented in the TFM as a threshold on $a_1$: $a_1 > a_1^\text {crit}$.

The second criterion is linked to the appearance of two maxima of the flux in the neighborhood of the tip, which is related to the value of the $a_3$ coefficient. When for a given radius $r_\text {B}$ the flux of the field has a single high maximum, the finger grows in the direction of this maximum. However, if the flux of the field from the sides of the tip becomes comparable to that from the front, or even higher (which corresponds to the bimodal field around the tip and the appearance of two maxima at $\pm\;\theta _0$—*Supplemental Information*, Fig. S1H), the finger attempts to grow in two directions at once, which results in a bifurcation. More precisely, we require the finger to split whenever $a_3/a_1$ becomes smaller than some critical value (which is negative if we consider the bimodal field around the tip or might be positive if we consider a single flat maximum^17^). This critical value depends on the value of $r_\text {B}$ (Eq. 6), which introduces a new length scale in the system. For an insightful discussion on this length scale in the case of river networks we refer to Ref.^10^.

Whenever a splitting criterion is fulfilled, two daughter branches are created at $\theta = \pm\;36^\circ$ (measured in the coordinate system around the tip as before). As shown in Refs^45,46,57^, the angle of $2\pi /5=72^\circ$ between the two daughter branches is the stable opening angle in the TFM. This characteristic opening angle has also been found in natural stream networks formed by groundwater seepage^9^ and has been used to characterize the climate dependence of river network patterns on Earth and Mars^58,59^.

## Results

### Forward evolution

Given the growth rules described above, we can follow the growth of the network starting from the initial positions of the branches (seeds) to understand how the growth rules impact the final geometry of the structure. However, except for the simplest cases of one- and two-finger solutions^46^, one needs to resort to numerical methods here.

First, we rescale the coordinates and the field in both the Laplace and the Poisson case (*Supplemental Information*, section 1), which results in the dimensionless equations:

9 $$\Delta \phi = 0\quad {\text{or}}\quad \Delta \phi {\text{ = }} - {\text{1}}$$

respectively. In both cases, the dimensionless growth law becomes:

10 $$\begin{aligned} v = (a_1)^\eta \,. \end{aligned}$$

Next, we construct a growth algorithm based on the finite element calculation of the driving field for a given geometry of the network and extension of the branches in streamline direction (*Supplemental Information*, section 2). The details of the growth algorithm are described in *Supplemental Information*, section 3.

**Figure 3:**
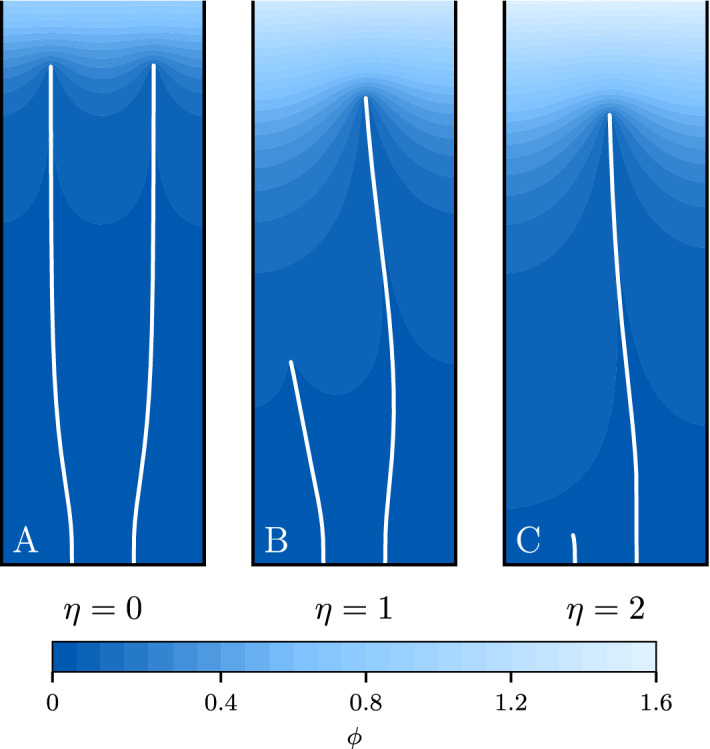
The evolution of two fingers for different growth exponents. Initially, the fingers are positioned symmetrically in the channel ($x_\text {initial} = \pm\;0.3$), but their heights differ (0.01 vs. 0.02). The units here are chosen in such a way that the channel extends from $x=-1$ to $x=1$. (**A**) For $\eta =0$ the fingers grow with the same velocity. (**B**,**C**) At larger $\eta$ the growth becomes unstable due to the competition between the fingers for an available flux. The colors in the figure correspond to the value of the field driving the growth.

**Figure 4:**
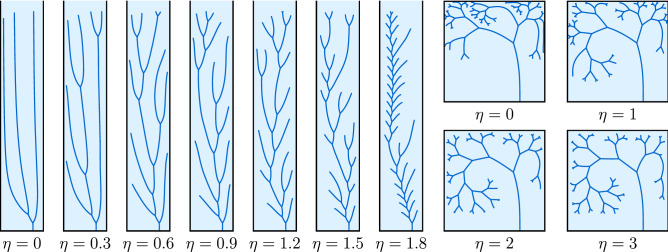
Comparison of the Laplacian and Poissonian networks for different $\eta$ exponents. On the left, Laplacian networks in a channel with reflective side walls and flux of the field coming from the top. On the right, Poissonian networks in a square box with reflective side and top walls and non-zero source term. In the latter case, each of the networks has the same total sum of the lengths of the branches. The networks grow from a single seed initially placed at 3/4 of the channel width. Fingers can bifurcate with the velocity criterion $a_1 > 0.8$ in the Laplacian case and with the bimodality criterion $a_3/a_1 < -0.1$ in the Poissonian case.

We begin by considering a simple case of two fingers growing in a long channel with constant flux of the field coming from the top ($(\nabla \phi )_{\vec {n}} = 1$), absorbing boundary conditions ($\phi =0$) on the fingers and the bottom wall, and reflective boundary conditions ($(\nabla \phi )_{\vec {n}} = 0$) on the side walls. The source term $P=0$ implies that the Laplace equation ($\Delta \phi = 0$) needs to be solved to calculate the field, hence the name of the networks obtained in such a setup—*Laplacian*. The aspect ratio of the channel was set at 1:25, with Figs. 3 and 4 showing only the lower portion of the domain.

For a single finger, the parameter $\eta$ would affect only the growth velocity, but not the network structure. The same holds for two identical fingers symmetrically placed left and right of the vertical symmetry line. Thus, we break the symmetry by starting from a configuration where the left finger is $50\%$ shorter, and hence collects slightly less flux than the right one. For $\eta =0$ the growth velocity of the fingers does not depend on the flux; thus, both fingers grow with the same velocity (Fig. 3A). For $\eta <0$ the growth process is stable and the velocity is inversely proportional to the flux. Thus, the shorter tip which collects less flux is growing faster, catching up with its longer sibling at some point. For $\eta >0$, however, we have a positive feedback between growth velocity and flux; thus, the right branch starts to outcompete the other. Eventually, it screens its sibling from the flux, just as the lightning rod screens the surrounding area. The larger the $\eta$, the stronger the effect, as shown in Fig. 3B,C.

Next, we present the evolution of branched networks, where we allow the splitting of the fingers according to the velocity criterion (Fig. 4). More specifically, a finger bifurcates if $a_1 > 0.8$. At low $\eta$, the competition between the fingers is relatively weak. It takes then a long time for one finger to outgrow the other sufficiently to intercept enough extra flux in order to split again. The bigger the $\eta$, the more dynamic the evolution with a larger number of tip splittings. Laplacian structures, similar to those presented in Fig. 4 can be observed in natural systems, such as corals^17^, dielectric breakdown patterns^23^, combustion fingers^39^, or leaf venation of evolutionary ancient plants^60^.

In a second series of simulations, we consider a non-vanishing source term $P \ne 0$. Now, the Poisson equation ($\Delta \phi = -1$) is solved in the domain, hence the name of the networks—*Poissonian*. We grow the networks in a square box with reflective boundary conditions on the top, left, and right walls. As before, the absorbing boundary conditions are imposed on the bottom wall and the network itself. Because the flux is now produced uniformly across the domain, the system does not have a preferred growth direction, contrary to the Laplacian case. As a tip splitting criterion, we have chosen the bimodality bifurcation rule ($a_3/a_1 < -0.1$). The two elements: (i) weaker competition between the fingers connected to a uniform distribution of the field sources and (ii) the bifurcation criterion based on the field on the sides of the tip result in the creation of fractal-like structures, with progressively shorter branches splitting in a self-similar way. Consequently, the geometry of Poissonian networks does not depend as strongly on the $\eta$ exponent as for Laplacian structures (compare the differences of trees in Fig. 4). Ramified, self-similar structures of this kind are encountered in river networks^10,22^ or blood vasculature^20,42^.

### The backward evolution algorithm

Having analyzed the deterministic forward growth, we now focus on the question of whether it is possible to recover the value of the growth exponent and the bifurcation criteria given only the final geometries of the networks, such as those shown in Fig. 4. To this end, we construct the Backward Evolution Algorithm. The idea of the method is to start with a set of possible growth exponents $\eta$, and then evolve the system back in time while collecting geometric information on shrinking structures. These data then allow us to assess which growth rule reproduces the evolution of the system in the most accurate way.

**Figure 5:**
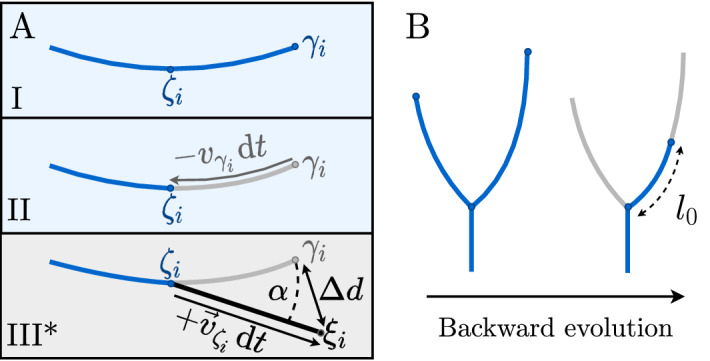
The Backward Evolution Algorithm. (**A**) I–II: The velocities of the tips ($\gamma _i$) are calculated, and each branch is trimmed by $v_{\gamma _i} \text {d}t$. This gives the previous tip positions $\zeta _i$, where we measure the local symmetry parameter ($a_2/a_1^2$). $\text {III}^*$: Additionally, we can use the forward algorithm to obtain an extrapolated position $\xi _i$, and two additional metrics: the overshoot ($\Delta d$—distance between $\gamma _i$ and $\xi _i$) and the angular deflection ($\alpha$). (**B**) Definition of the length mismatch ($l_0$) at a bifurcation during the backward evolution.

To be more specific, we first assume some value of $\eta = \eta ^*$, and then calculate the velocities at the current positions of the tips ($v_{\gamma _i}$—Fig. 5A, panels I–II), as well as the distance over which each tip will move over a timestep $\text {d}t$: $\text {d}s_i(t) = v_{\gamma _i} \text {d}t = (a_1^i)^{\eta ^*} \text {d}t$. Using the reversed version of the growth algorithm (*Supplemental Information*, section 3) we then obtain the projected previous position of the tip ($\zeta _i$) and trim each branch accordingly. This procedure can be repeated iteratively, trimming progressively more and more segments of the branches, and thus shrinking the whole network.

In each step of the BEA we collect the local symmetry measure—the $a_2/a_1^2$ value. As mentioned earlier, the $a_2$ coefficient should vanish along the real trajectory of a growing tip. Thus the value of $a_2$ (rescaled by $a_1^2$ to make it dimensionless) is a convenient measure of how far we are from the real trajectory of the finger. Additionally, we make a virtual forward step obtaining an extrapolated position of the tips ($\xi _i$ in Fig. 5A, panel $\text {III}^*$). Based on these data, the overshoot ($\Delta d$—distance between the points $\gamma _i$ and $\xi _i$) and the angular deflection ($\alpha$—angle between $\gamma _i$ and $\xi _i$) are calculated. In this way, a backward-forward method is incorporated into the BEA, collecting the metrics throughout the whole backward evolution of the network, and not only in a single backward-forward step (as opposed to Ref^11^).

We evolve the network backward in time until it vanishes entirely. In this manner, in each step of the BEA we collect *N* values for each metric, where *N* is the number of tips. Next, we calculate the quartiles ($Q_1$, *M*, $Q_3$—quantiles of order 25%, 50% (median) and 75%, respectively) of the data collected over all time steps. This procedure is repeated for a range of $\eta ^*$ values. We expect that all metrics (local symmetry, overshoot and angular deflection) will be minimized at a particular value of $\eta ^*$, which should correspond to the growth exponent of the original network ($\eta _0$).

**Figure 6:**
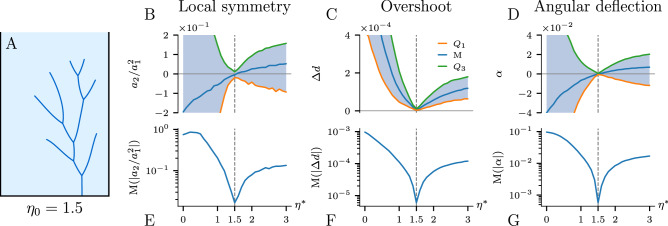
The Backward Evolution Algorithm applied to a synthetic Laplacian network. (**A**) Original network created with $\eta _0 = 1.5$ to which the algorithm was applied. (**B**–**D**) Quartiles of the collected values of the local symmetry, overshoot, and angular deflection. (**E**–**G**) Median of the absolute value of the corresponding metric plotted in a logarithmic scale. The pronounced minima allow us to estimate the correct $\eta _0$ (marked with the black dashed line on each plot). The results show that the BEA can precisely reconstruct the growth exponent of a given network.

To examine the effectiveness of the BEA, we first grow a test network with some specific value of $\eta = \eta _0$. Then we evolve it backward in time for a range of different $\eta ^*$ values and plot the resulting metrics as a function of $\eta ^*$. Figure 6 shows the plots for a Laplacian network originally grown with $\eta _0=1.5$. Figure 6B–D presents the quartiles of the collected data. We observe that each median approaches zero exactly at the $\eta _0$ value that was initially imposed to produce the network (marked with the black dashed line).

Note that at the correct value of the exponent ($\eta ^\star =\eta _0$) the interquartile range (IQR—distance between $Q_1$ and $Q_3$ in 6B–D) of metrics collected during the backward evolution approaches zero. Conversely, for an incorrect $\eta ^*$, the distribution of the collected data set has some spread due to the fact that we land in different places around the initial position after the backward-forward step. Consequently, the IQR dependence also exhibits a minimum at the correct $\eta ^*=\eta _0$ value. In Fig. 6E-G we show the median of the absolute value of the collected data in a logarithmic scale. Pronounced minima observed at $\eta ^*=\eta _0$ reflect both the fact that the value of a given metric is minimal for the correct $\eta ^*$ and the fact that the variance of the metric is minimal at $\eta ^*=\eta _0$.

**Figure 7:**
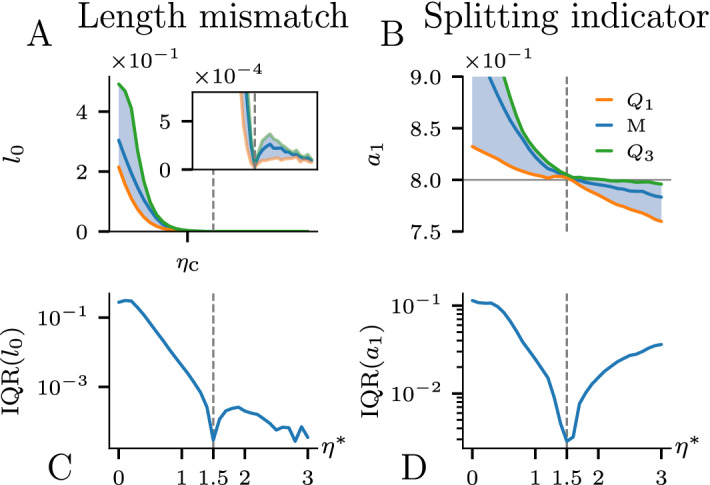
Analysis of the bifurcation points in the BEA: length mismatch ($l_0$) and $a_1$ coefficient at the splitting point as a function of the growth exponent ($\eta ^*$). (**A**–**B**) Quartiles of the values of a corresponding metric. (**C**–**D**) Interquartile range of the distributions (distance between $Q_1$ and $Q_3$). The results confirm that the BEA is capable of reconstructing not only the growth exponent of a given network based on its bifurcation points but also the mechanism behind the tip splitting.

Let us consider the possibility of using the position of the bifurcation points in the solution of the inverse problem. Since the fingers split at a specific point, then—with the use of the correct growth rule—they should also converge to the same bifurcation point as the network is grown backward. If they do not converge simultaneously, then measuring the excess length of the longer branch (length mismatch $l_0$ in Fig. 5B) and minimizing it with respect to $\eta ^*$ should allow one to recover the correct growth exponent. Indeed, if we start with $\eta ^*=0$, the branches will be trimmed at the same rate and it will take less time for the shorter branch to reach the bifurcation point. In such a case, the length mismatch will be exactly equal to the initial length difference between the branches. With $\eta ^*$ approaching $\eta _0$, the length mismatch decreases monotonically to zero, since the longer branch—which was growing faster—will also be trimmed faster in the backward evolution. At the correct $\eta ^*=\eta _0$ the branches converge to the bifurcation point simultaneously, which should result in the minimum of the length mismatch, as well as its interquartile range. Throughout the backward evolution, we collect the mismatch value from all bifurcation points. The corresponding median and interquartile range exhibit minima at the correct $\eta ^*=\eta _0$, as shown in Fig. 7A,C.

Interestingly, we observe a dramatic decrease of the length mismatch above $\eta ^* \approx 1$ (Fig. 7A). This behavior is related to the stability of a single bifurcation in an unbounded domain, as studied analytically by Carleson and Makarov^45^. They have shown that for $\eta < \eta _c \approx 1.09382$ the growth of the daughter branches after splitting is stable and that the fingers move away from each other with equal velocities. For $\eta > \eta _c$, the competition between the branches makes their growth unstable, with one speeding up and the other slowing down. With a flipped time arrow, the stability of the system is also reversed. Thus, for $\eta > \eta _c$ both daughter branches should reach the bifurcation point at the same time, hence almost zero length mismatch for larger growth exponents. After zooming in we nevertheless observe a minimum at the correct $\eta ^*$, which is related to the higher order effects such as the presence of other branches and the influence of the boundaries of the system. The above reasoning does not hold for bifurcations triggered by the bimodality criterion in the Poissonian case. Here, due to the presence of local sources, the competition between branches is weaker and, hence, a bifurcation can grow in a stable manner even for relatively high $\eta$. On the contrary, in the backward evolution there is no stabilizing effect, and we observe a pronounced minimum of the length mismatch (*Supplemental Information* Fig. S2E).

One can also analyze the expansion coefficients of the field indicating a splitting event at the bifurcation point ($a_1$ for the velocity bifurcation criterion or $a_3/a_1$ for the bimodality criterion). As can be seen in Fig. 7B, the values of $a_1$ at the bifurcation points are converging to one value for the correct $\eta ^*=\eta _0$. Hence, minimizing the interquartile range of the bifurcation indicators is another way to estimate $\eta _0$ (Fig. 7D). Importantly, the values of $a_1$ or $a_3/a_1$ converge exactly to the values used as bifurcation criterion thresholds when the network was originally grown. Thus, the BEA analysis of bifurcations gives us not only the correct growth exponent but also allows us to recover the bifurcation criterion for a particular network.

The BEA can be applied as well to a Poissonian tree, giving similarly precise estimates of the growth exponent (*Supplemental Information*, Fig. S2). The precision of the predictions decreases somewhat with an increasing growth exponent, as shown in *Supplemental Information*, Fig. S3. This is connected to the increasing growth instability at high $\eta$—with increasing growth exponent the minima of the metrics become less pronounced, finally flattening totally, which makes the reconstruction of the growth rules and estimation of $\eta _0$ increasingly harder. This effect becomes pronounced around $\eta _0=4$ for the Laplace case and $\eta _0=6$ in the Poisson case. The wider range of precision in the Poissonian case might be the result of weaker screening and smaller differences of velocities between the fingers than in the corresponding Laplacian case.

### Backward evolution of the river network

As a final application of our model, we use it to assess the growth laws of a real river network, namely the White River basin (HUC-01080105) in central Vermont, USA. This river network grows in a humid environment, where diffusive fluxes and groundwater flows may play a crucial role in its formation^58,61–63^. For our analysis, we used medium resolution channels in the White River basin, as mapped by the NHDPlus dataset^7^ and preprocessed as described in *Supplemental Information*, section 4. We assume that the field driving the growth can then be described by the Poisson equation (Eq. 2), with precipitation being responsible for the source term. Using Eq. (7) for the rate of erosion, we can apply the BEA to extract the parameters of the underlying growth law.

**Figure 8:**
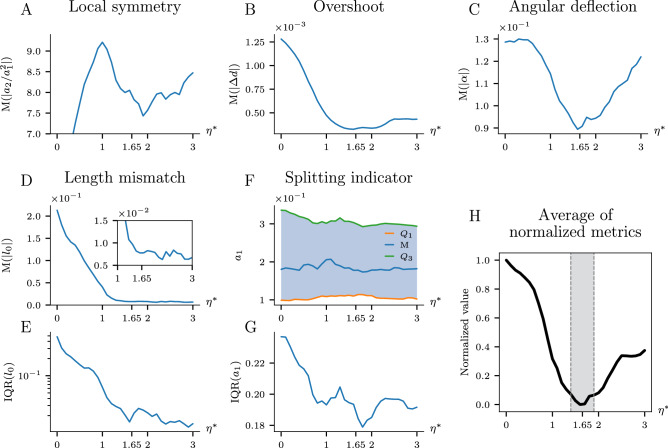
Results of the BEA on the White River network in Vermont, USA. (**A**–**C**) Median of the absolute value of the local symmetry, overshoot, angular deflection (**D**–**E**) Length mismatch (median of the absolute value and IQR) (**F**–**G**) Splitting indicator (quartiles and IQR) (**H**) Average of normalized metrics with a minimum around $\eta ^*=1.65\pm 0.25$ (marked on each subplot).

The results of our analysis are presented in Fig. 8. As one could anticipate, the metrics are much more noisy than the ones based on the artificial network, with less pronounced minima of overshoot and angular deflection and only a local minimum of the local symmetry in the region where the rest of the metrics indicate $\eta _0$. The bifurcation length mismatch plot (Fig. 8B) is less revealing, with several shallow minima for $\eta >1$.

To determine the growth exponent, we make use of the multiple metrics included in the BEA (local symmetry, overshoot, angular deflection Fig. 8A–C; their IQR *Supplemental Information*, Fig. S5; length mismatch and its IQR Fig. 8D–E; and IQR of the splitting indicator Fig. 8G). We normalize each metric so that its values, as a function of $\eta ^{\star }$, range from 0 to 1 and average the resulting functions to produce a final measure of the fit of a particular $\eta$ value (Fig. 8H). The resulting function has a single minimum at $\eta ^*=1.65\pm 0.25$.

The appearance of the minimum in the splitting indicator plot (Fig. 8G) around $\eta ^*=1.65$ suggests that in this case the branch splitting events are triggered by an increase of the tip growth velocity. This is further confirmed by the analysis of the histogram of branch lengths in the White River, which shows an exponential distribution (*Supplemental Information*, section 5) similar to the analogous distributions for synthetic networks grown with the velocity bifurcation criterion. Additionally, we observe an abrupt decrease of the length mismatch for $\eta ^*>\eta ^\text {c}$ (8d), which is another indicator of the velocity bifurcation criterion, as already discussed (compare Fig. 7A and *Supplemental Information*, Fig. 2E).

## Summary

We have shown that it is possible to use the final geometry of the spontaneously grown network to decipher its growth dynamics using the Backward Evolution Algorithm. We tested it on several synthetic networks and then analyzed the natural system—the White River basin. The BEA metrics consistently suggest that the growth exponent of this network is around $\eta = 1.65 \pm 0.25$. This indicates a nonlinear relation between the erosion rate and diffusive flux coming to springs, implying strong competition between different tributaries for groundwater flow. Additionally, we determined that the splitting events in this network were triggered by an increase of the growth velocity of the channel heads. The BEA method should be equally applicable to other systems, such as leaf venation, blood vessel networks, or dielectric breakdown patterns.

## Supplementary Information

Supplementary Information.

## Data Availability

The datasets used and/or analyzed during the current study together with the source code are available on the GitHub repository (https://github.com/stzukowski/reticuler).
