# Supplementary material for: Through history to growth dynamics: deciphering the evolution of spatial networks
Source: Sci Rep. 2022 Nov 27;12:20407. doi: 10.1038/s41598-022-24656-x (PMC9701698; doi:10.1038/s41598-022-24656-x)
Supplement: Supplementary file 1 — Supplementary Information. [file 41598_2022_24656_MOESM1_ESM.pdf]

# *Supplemental Material*
# Through history to growth dynamics: deciphering the evolution of spatial networks

**Stanisław Żukowski**[1,2], **Piotr Morawiecki**[3], **Hansjörg Seybold**[4], **and Piotr Szymczak**[1,*]

[1]Institute of Theoretical Physics, Faculty of Physics, University of Warsaw, Warsaw, Poland
[2]Laboratoire Matière et Systèmes Complexes (MSC), UMR 7057, CNRS & Université Paris Cité, Paris, France
[3]Department of Mathematical Sciences, University of Bath, Bath, United Kingdom
[4]Department of Environmental System Science, ETH Zürich, Zürich, Switzerland
[*]piotr.szymczak@fuw.edu.pl

## 1 Rescaling the Equations

First, we make the Poisson equation dimensionless by rescaling both the coordinates and the field as follows: $x' = \frac{x}{w}$ (with $2w$ being the width of the system) and $\phi' = \frac{\kappa}{w^2 P}\phi$. This leads to:

$$\Delta'\phi' = -1. \tag{1}$$

Next, we cast the growth rate equation (Eq. 8 in the main text) in the dimensionless form by scaling time by $t' = \sigma w^{\frac{3}{2}\eta - 1}(\frac{P}{\kappa})^{\eta} t$ to obtain:

$$v' = (a_1')^{\eta}\,, \tag{2}$$

where $a_1' = \frac{\kappa}{w^{3/2}P}a_1$.

The rescaling in the Laplacian case ($P = 0$) is slightly different. Here, the system is usually fed by a flux of the field coming from the outside boundary of the system ($J_0$), so $P$ in the Poissonian scalings is replaced by $J_0/w$. Now, $t'' = \sigma w^{\frac{1}{2}\eta - 1}(\frac{J_0}{\kappa})^{\eta} t$, and the field: $\phi'' = \frac{\kappa}{wJ_0}\phi$, which leads to:

$$\Delta''\phi'' = 0\,, \tag{3}$$

$$v'' = (a_1'')^{\eta} \tag{4}$$

with $a_1'' = \frac{\kappa}{w^{1/2}J_0}a_1$.

## 2 Trajectory of the Tip

To construct a precise growth algorithm, we first derive the trajectory of the finger in the neighborhood of the tip. It can be obtained using the fact that the growth proceeds along a unique streamline going through the tip[1]. To simplify the analysis, let us move to the complex plane with $z = x + iy$ and analyze the solution of the Laplace equation ($\Delta\phi = 0$) in the vicinity of the tip. It is convenient to introduce the complex potential $\Phi(z)$ such that $\Phi = \phi + i\psi(z)$, with $\psi(z)$ being the corresponding stream function. Additionally, let us direct the finger along the negative imaginary axis with the tip at the origin. To find the complex potential near the finger tip, we first map the area outside the finger to the upper half plane by the mapping $\omega = \sqrt{iz}$. In the $\omega$ plane (mathematical plane), the complex potential vanishing at the real axis can be expanded in the Taylor series:

$$\tilde{\Phi}(\omega) = -i(a_1\omega + a_2\omega^2 + a_3\omega^3 + \dots)\,. \tag{5}$$

This solution can then be mapped back to the $z$ plane (physical plane) to yield:

$$\Phi(z) = \tilde{\Phi}(\omega(z)) = -i(a_1(iz)^{1/2} + a_2 iz + a_3(iz)^{3/2} + \dots)\,, \tag{6}$$

which is equivalent to Eq. (4) in the main text.

To determine the streamlines in the mathematical plane, where $\omega = u + iv$, we calculate $\tilde{\psi}(u,v)$:

$$\tilde{\psi}(u,v) = \mathrm{Re}[\tilde{\Phi}(u+iv)] = -a_1 u - a_2 u^2 + a_2 v^2 + \dots\,, \tag{7}$$

and equate it to a constant:

$$\tilde{\psi}(u,v) = A \implies -a_1 u - a_2 u^2 + a_2 v^2 = A\,. \tag{8}$$

The curves describing the streamlines, $\tilde{\gamma}(u,v)$, can thus be parameterized only with one parameter $\tau$, with $u = \tau$ and $v = i(\tau - \tilde{\gamma}(u,v))$:

$$-a_1 u - a_2\tau^2 + a_2 i^2(\tau - \tilde{\gamma})^2 = A \implies \tilde{\gamma}(\tau) = \tau + i\sqrt{\beta\tau + \tau^2 + A/a_2}\,, \tag{9}$$

where $\beta = a_1/a_2$. As the tip is mapped to the point $\omega = 0$ in the mathematical plane, the streamline of interest is then given by $\tilde{\gamma}_0(0) = 0$, thus:

$$\tilde{\gamma}_0(\tau) = \tau + i\sqrt{\beta\tau + \tau^2}\,. \tag{10}$$

Moving back to the physical plane (with $z = -i\omega^2$) we obtain the formula describing the streamline entering the tip[2]:

$$\gamma_0(\tau) = -i\tilde{\gamma}_0(\tau)^2 = 2\tau\sqrt{\beta\tau + \tau^2} + i\beta\tau\,. \tag{11}$$

Using the fact that $x = \mathrm{Re}[\gamma_0(\tau)] = 2\tau\sqrt{\beta\tau + \tau^2}$ and $y = \mathrm{Im}[\gamma_0(\tau)] = \beta\tau$ we obtain:

$$x(y) = 2\sqrt{\frac{y^3}{\beta^2} + \frac{y^4}{\beta^4}} \approx \frac{2}{\beta} y^{3/2}\,. \tag{12}$$

We can also calculate the arclength as a function of $y$:

$$s(y) = \int_0^y \sqrt{1 + (\frac{\mathrm{d}x}{\mathrm{d}y'})^2}\mathrm{d}y' = \int_0^y \sqrt{1 + \frac{9}{\beta^2}y'}\mathrm{d}y' = \frac{2\beta^2}{27}\left[(\frac{9}{\beta^2}y + 1)^{3/2} - 1\right]\,. \tag{13}$$

Finally, we get the following set of equations for the streamline ($\beta = a_1/a_2$):

$$y(s) = \frac{\beta^2}{9}\left[\left(\frac{27s}{2\beta^2} + 1\right)^{2/3} - 1\right]\,, \tag{14}$$

$$x(y) = 2\sqrt{\frac{y(s)^3}{\beta^2} + \frac{y(s)^4}{\beta^4}}\,. \tag{15}$$

The above reasoning was carried out for the Laplacian case. However, as discussed in the main text, the Poissonian case will be fully equivalent, since we are interested in the small neighborhood of the tip here. In such a small area, the flux from the local sources is negligible compared to the flux from the regions far away from the tip, which brings the description of the system to the Laplacian case with flux entering through the boundaries only.

## 3 Growth Algorithm

Our growth algorithm is composed of a finite element solver[3] for the Laplace or Poisson equation, and an integration step extending the tips along the streamline $\gamma_0$ (Eq. (11)) by $\mathrm{d}s = v\mathrm{d}t$ via Eqs. (14)-(15). The second order method allows for an increased accuracy in the network growth simulations in comparison to the previous models[2,4].

The velocity of the tip is derived from the field through the expansion coefficients $a_1$, $a_2$ and $a_3$, which can be obtained by integration over a small circle of radius $r$:

$$a_1 = \frac{1}{\pi r_0^{1/2}}\int_{-\pi}^{\pi}\phi(r_0,\theta)\cos\frac{\theta}{2}\mathrm{d}\theta\,, \tag{16}$$

$$a_2 = \frac{1}{\pi r_0}\int_{-\pi}^{\pi}\phi(r_0,\theta)\sin\theta\mathrm{d}\theta\,, \tag{17}$$

$$a_3 = \frac{1}{\pi r_0^{3/2}}\int_{-\pi}^{\pi}\phi(r_0,\theta)\cos\frac{3\theta}{2}\mathrm{d}\theta\,. \tag{18}$$

Importantly, we require the numerical method used to construct the tip trajectory to be reversible. This ensures that after the backward-forward step in the Backward Evolution Algorithm, we end up in the same point, provided that a correct growth rule is used (except for the effects of numerical noise). Keeping this in mind, we use the implicit trapezoidal method[5]:

$$\vec{r}(t+\mathrm{d}t) = \vec{r}(t) + \frac{1}{2}\left[\mathrm{d}\vec{r}(t) + \mathrm{d}\vec{r}(t+\mathrm{d}t)\right], \tag{19}$$

which we solve iteratively. The reversed version of this algorithm can be expressed as follows:

$$s_i(t-\mathrm{d}t) = s_i(t) - \frac{1}{2}\left[\mathrm{d}s_i(t) + \mathrm{d}s_i(t-\mathrm{d}t)\right]. \tag{20}$$

Additionally, to limit the maximum displacement of the tip, we adjust the value of $\mathrm{d}t$ at each time step to keep $\mathrm{d}s$ of the fastest branch constant.

We tested our algorithm on a simple case of one finger growing in a semi-infinite channel with flux of the field coming from infinity. For this case, the analytical solution has been derived[6] which was compared with our numerical solutions to validate it.

## 4 Preprocessing of the river network

The data was extracted from the NHDPlus dataset[7]. Then, to limit the computational cost, we selected seven sub-trees of the whole network to apply the BEA algorithm on them (Fig. 4). The fragments were chosen to stem from the highest Strahler order streams[8], which was also treated as an absorbing boundary of their growth domain. The rest of the boundaries were marked out so that they lay between the neighboring sub-trees, following the watershed lines (see Fig. 4B). On these boundaries reflecting boundary conditions are imposed. The setup is thus similar to the box where the Poissonian simulations from Fig. 4 in the main text were conducted, but with more complicated geometry of the domain. Before applying the BEA algorithm, we also smoothed out the branches. We performed the sensitivity analysis introducing small changes in the shape of the selected sub-domain and smoothing procedure, changing the time step; and did not notice any significant difference in the results. The plots presented in Fig. 8 in main text and Fig. 5 are averaged results from the seven different areas of the White River basin.

## 5 Distribution of Segment Lengths

In Fig. 6 we present a comparison of histograms of segment lengths (distances between bifurcations) for: (i) the entire White River basin, (ii) simulations with the velocity-based splitting criterion and (iii) simulations with the bimodality splitting criterion. In the first two cases, we observe exponential distribution of segment lengths. However, there is a deficiency of very short branches (<500m for White River and <0.6 for the simulation). This is because the flux that was prior to bifurcation coming to a single mother branch is now divided between two daughter branches. It takes then some time for the new branches to move away from each other enough to get sufficient velocity and split again, which results in a lower number of very short segments. By contrast, simulations with the bimodality splitting criterion do not exhibit these features.

## References


**1.** Devauchelle, O. *et al.* Laplacian networks: Growth, local symmetry, and shape optimization. *Phys. Rev. E* **95**, 033113 (2017).

**2.** Petroff, A. P., Devauchelle, O., Seybold, H. J. & Rothman, D. H. Bifurcation dynamics of natural drainage networks. *Philos. Transactions Royal Soc. A* **371**, 20120365 (2013).

**3.** Hecht, F. New development in FreeFem++. *J. Numer. Math.* **20**, 251–266 (2012).

**4.** Cohen, Y. *et al.* Path selection in the growth of rivers. *Proc. Natl. Acad. Sci.* **112**, 14132–14137 (2015).

**5.** Iserles, A. *A First Course in the Numerical Analysis of Differential Equations* (Cambridge University Press, 2009).

**6.** Gubiec, T. & Szymczak, P. Fingered growth in channel geometry: A Loewner-equation approach. *Phys. Rev. E* **77**, 041602 (2008).

**7.** McKay, L. *et al.* NHDPlus version 2: User guide (2012).

**8.** Horton, R. E. Erosional development of streams and their drainage basins; hydrophysical approach to quantitative morphology. *Geol. Soc. Am. Bull.* **56**, 275–370 (1945).

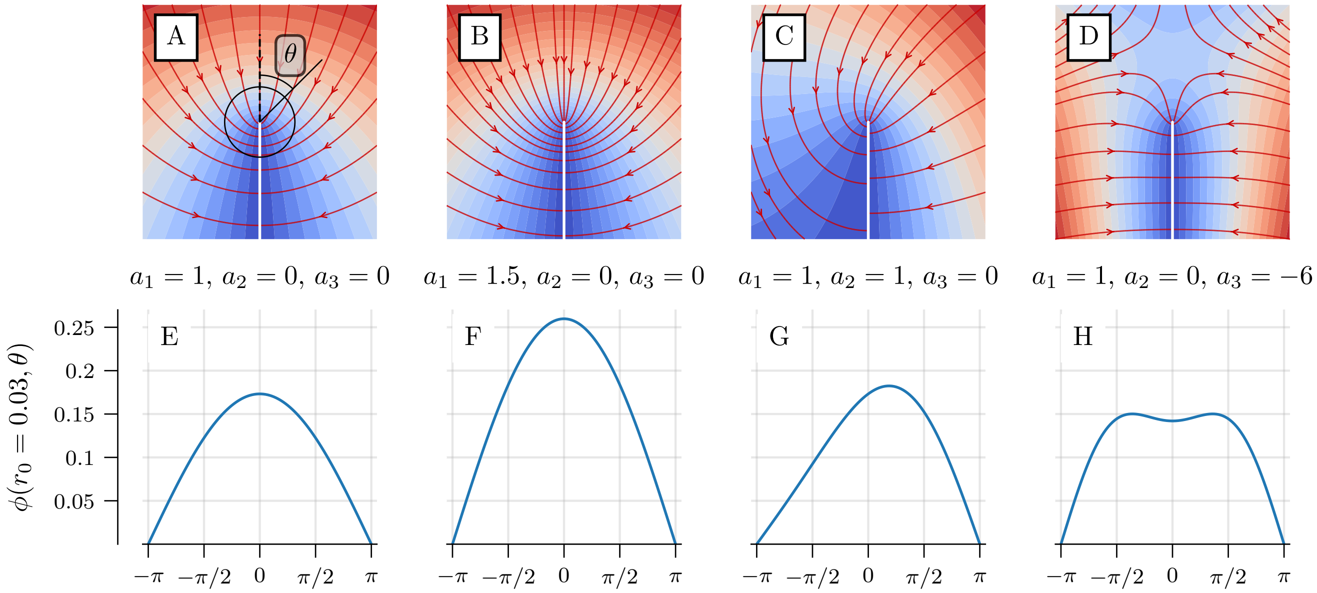


**Figure 1.** Impact of the $a_1, a_2, a_3$ coefficients on the field around the finger tip (after Ref.[2]). (A-D) Field around the finger tip (indicated by a white line). Colors from red to blue are related to the field magnitude, red arrows follow the field lines. (E-H) Profiles of the field along a small circle around the tip (black circle in A).

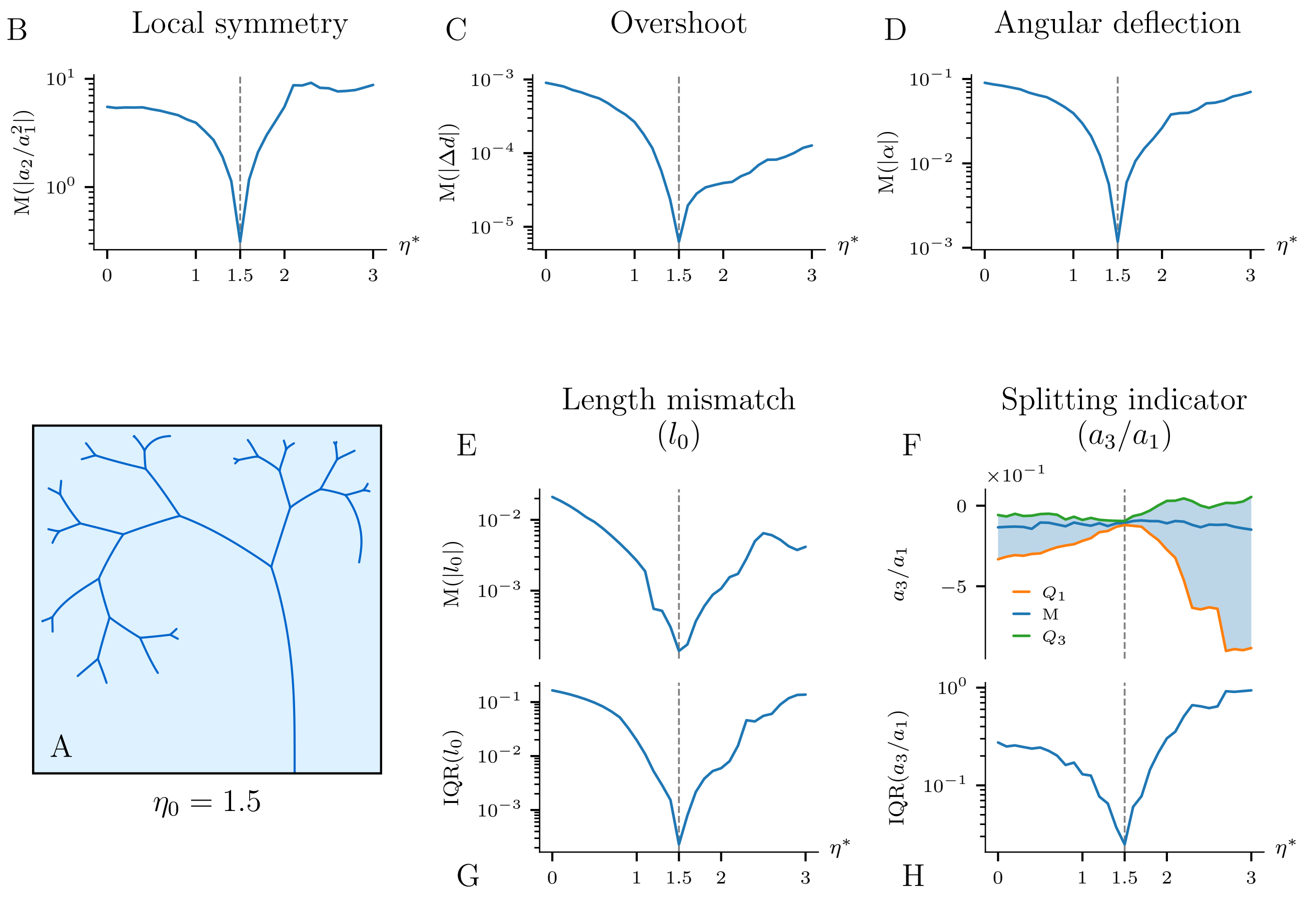


**Figure 2.** The Backward Evolution Algorithm on the synthetic Poissonian network. (A) A Poissonian network created with $\eta_0 = 1.5$, on which the algorithm was applied. (B-D) Median of the absolute value of local symmetry parameter, overshoot and angular deflection plotted in logarithmic scale. Pronounced minima allow us to estimate the correct $\eta_0$ (marked with the black dashed line on each plot). (E) Median of the absolute value of the length mismatch plotted in logarithmic scale. (F) Quartiles of the values of the bifurcation indicator $a_3/a_1$. (G-H) Interquartile range of the distributions (distance between $Q_1$ and $Q_3$) of the metrics from the bifurcation points.

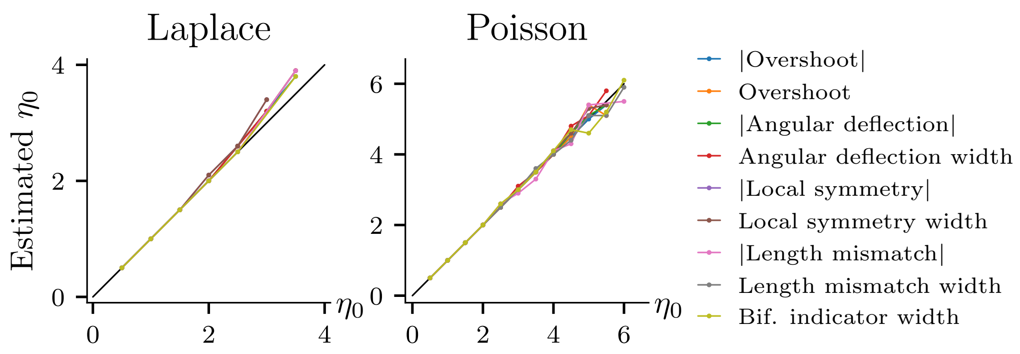


**Figure 3.** Reliability of the BEA in the Laplace and Poisson cases

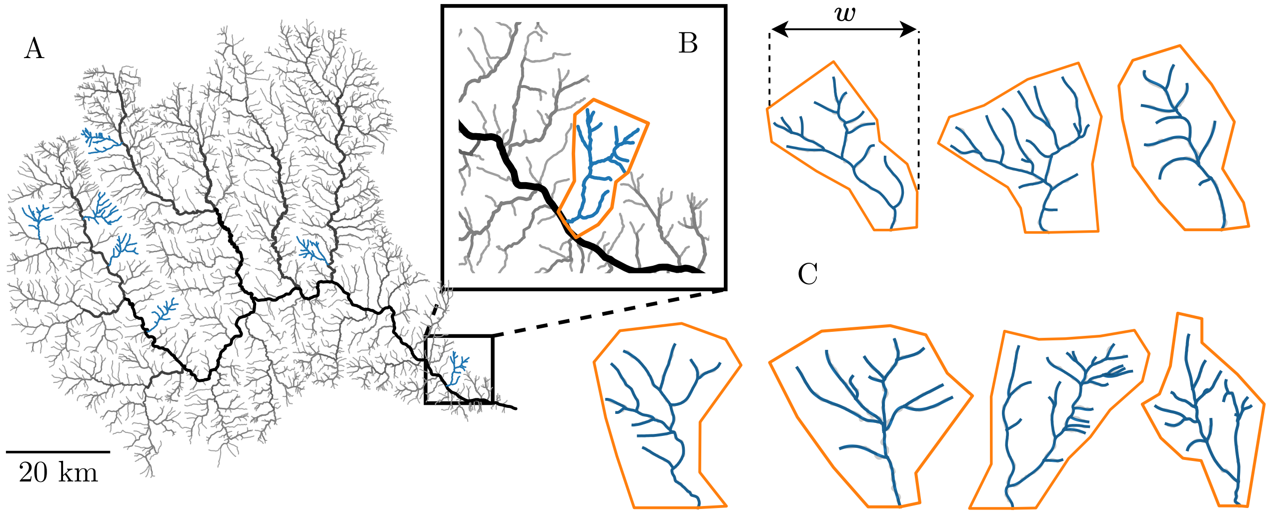


**Figure 4.** Preprocessing of the White River network before applying the BEA. (A) Whole White River basin with seven selected sub-trees marked in blue. (B) Zoom in on one of the selected sub-trees stemming from the highest Strahler order stream (black thick line). The rest of the boundaries (orange) were marked out so that they lay between the neighboring sub-trees. (C) Seven selected sub-trees with their domains after smoothing (blue). The original shapes are plotted in gray. For the numerical analysis, each of the sub-trees was rescaled to the same width $w = 2$.

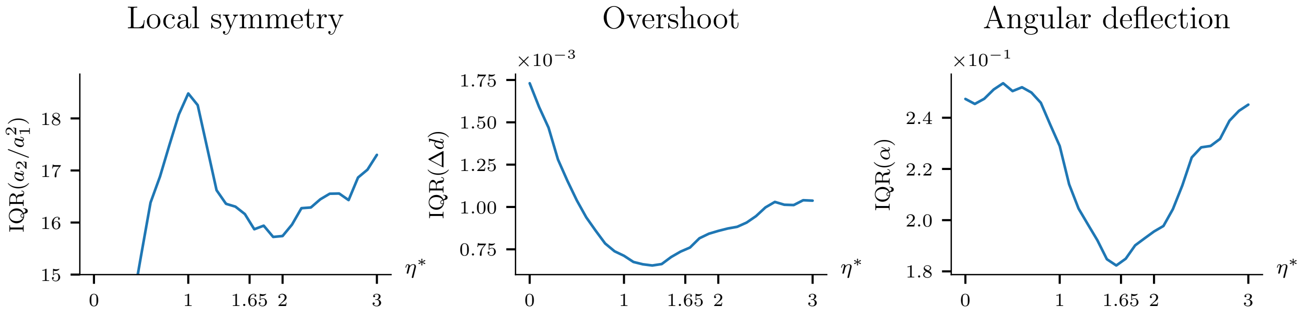


**Figure 5.** Interquartile range of the distributions of the local symmetry parameter, overshoot and angular deflection collected in the analysis of White River, Vermont, USA

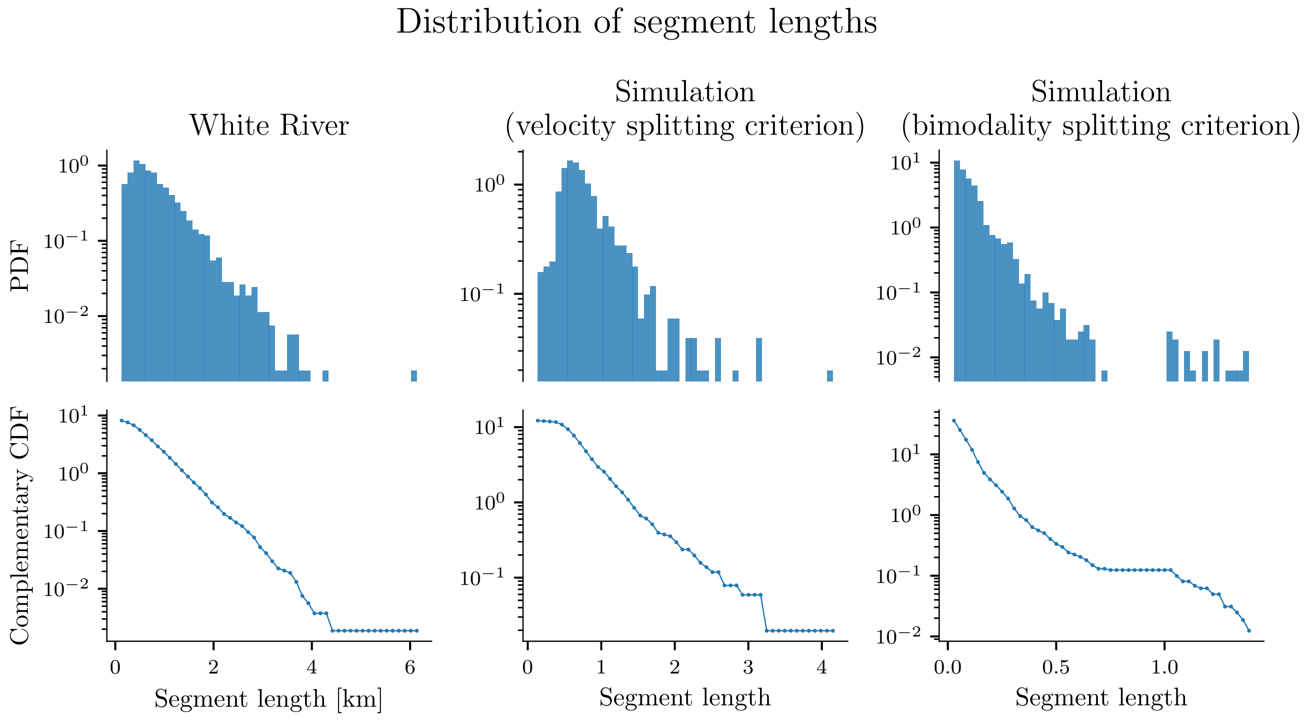


**Figure 6.** Comparison of the distributions of segment lengths in White River, Vermont, USA, a simulation with the Poissonian driving field and velocity-based splitting criterion, and a Poissonian simulation with the bimodality splitting criterion. The first row presents the probability density function of the segment lengths, whereas the second row shows complementary cumulative distribution functions.
